# Impact of Imperfect Test Sensitivity on Determining Risk Factors: The Case of Bovine Tuberculosis

**DOI:** 10.1371/journal.pone.0043116

**Published:** 2012-08-13

**Authors:** Camille Szmaragd, Laura E. Green, Graham F. Medley, William J. Browne

**Affiliations:** 1 School of Veterinary Sciences, University of Bristol, Langford, Devon, United Kingdom; 2 School of Life Sciences, University of Warwick, Coventry, West Midlands, United Kingdom; Queen’s University Belfast, United Kingdom

## Abstract

**Background:**

Imperfect diagnostic testing reduces the power to detect significant predictors in classical cross-sectional studies. Assuming that the misclassification in diagnosis is random this can be dealt with by increasing the sample size of a study. However, the effects of imperfect tests in longitudinal data analyses are not as straightforward to anticipate, especially if the outcome of the test influences behaviour. The aim of this paper is to investigate the impact of imperfect test sensitivity on the determination of predictor variables in a longitudinal study.

**Methodology/Principal Findings:**

To deal with imperfect test sensitivity affecting the response variable, we transformed the observed response variable into a set of possible temporal patterns of true disease status, whose prior probability was a function of the test sensitivity. We fitted a Bayesian discrete time survival model using an MCMC algorithm that treats the true response patterns as unknown parameters in the model. We applied our approach to epidemiological data of bovine tuberculosis outbreaks in England and investigated the effect of reduced test sensitivity in the determination of risk factors for the disease. We found that reduced test sensitivity led to changes to the collection of risk factors associated with the probability of an outbreak that were chosen in the ‘best’ model and to an increase in the uncertainty surrounding the parameter estimates for a model with a fixed set of risk factors that were associated with the response variable.

**Conclusions/Significance:**

We propose a novel algorithm to fit discrete survival models for longitudinal data where values of the response variable are uncertain. When analysing longitudinal data, uncertainty surrounding the response variable will affect the significance of the predictors and should therefore be accounted for either at the design stage by increasing the sample size or at the post analysis stage by conducting appropriate sensitivity analyses.

## Introduction

The estimation of disease incidence and prevalence, and the identification of potential risk factors associated with a disease are hampered by imperfect diagnostic tests. While the imperfect nature of tests is widely acknowledged, and several methods have been devised to account for imperfect test sensitivity and specificity when estimating disease incidence and prevalence (e.g. [1]–[3]), the impact of imperfect testing on the determination of risk factors has rarely been directly studied. The methods proposed to correct for imperfect testing have generally been based on sensitivity analyses and produce adjusted prevalence estimates for specific scenarios. This is a valid approach for cross-sectional studies, but ignores the implications that the test result of an individual subject (or unit) might affect the testing regime and the subsequent tests performed on the same subject/unit in a longitudinal setting. In this paper, we consider the use of discrete time survival models [4] to study risk factors for disease diagnosis in a dynamic context (i.e. where the status of an individual unit at a point in time is dependent on the status at the previous time point), and propose a novel extension to the standard model to handle imperfect tests using Monte Carlo Markov chain (MCMC) methods. The methods proposed can be used in many infectious disease scenarios but here we focus on modelling of risk factors for bovine tuberculosis (bTB) in Great Britain using a subset of data from the Randomised Badger Culling Trial (RBCT) [5]. There are many potential approaches for modelling this dataset and the purpose of the current work is not to propose a definitive best fitting procedure but to examine the impact of test sensitivity on one particular approach by adjusting for the imperfect sensitivity of the currently used test to define exposure to bovine tuberculosis, the single intra-dermal comparative cervical tuberculin (SICCT) test.

Bovine tuberculosis is a major economic problem for the British cattle livestock industry, which has seen a continuous increase in the disease incidence rate over the past 25 years [6]. Incidence and prevalence of bTB is generally reported in terms of numbers of new herd break downs (HBDs) per area, where a HBD occurs if at least one animal in a herd tests positive when the herd is not currently known to be infected. The number of HBDs is closely linked to the testing regime and in Great Britain (GB); routine testing of a cattle herd is conducted at a frequency determined by the prevalence of bTB in the parish as observed by the testing regime [7]. The guidelines for testing are in theory quite clear: during routine testing, all qualifying animals in the herd are tested using the SICCT test. Three days after testing, the test is read and if at least one animal is a reactor (i.e. the animal has reacted positively to the test), the herd is placed under movement restrictions; all reactors are compulsorily slaughtered and subject to *post mortem* examination. This event is known as a HBD. Results of the *post-mortem* examination and/or laboratory culture of tissues lead to the HBD being either classified as confirmed (evidence of *M. bovis* found) or not [8]. Herds in which reactor animals are confirmed are re-tested at a minimum interval of 60 days until they have had two consecutive clear tests. They are re-tested again after a minimum interval of 6 months and again after a further 12 months. All reactors removed prior to a second clear 60 day test are attributed to the breakdown incident and animal movement restrictions remain in place throughout the period [9]. Herds in which *M. bovis* is not confirmed in any reactor are also placed under restrictions and re-tested after 42 days. If this test is clear, restrictions are lifted and the herd returns to the routine testing cycle. Confirmation of a HBD also triggers testing of contiguous herds and herds from which reactor animals have been purchased during the period extending back to two months before the previous clear herd test. Any animals sold by the breakdown farm to other farms during this same period are also tested. The testing regime is thus designed to follow possible paths of transmission to neighbouring herds or through traded cattle to attempt to contain the infection, as well as to provide surveillance data.

In the literature, numerous risk factors associated with cattle HBDs have been identified and can be categorised into cattle- or herd-related factors (including cattle movements) and wildlife (e.g. badger) factors [9]–[17]. The risk factors identified vary between studies according to the data available and statistical approaches used. Little attention has so far been given to the effect that the imperfect nature of the tuberculin test has on the significance of the risk factors. While the specificity of the test is generally accepted to be close to 100% (i.e. there are very few false positive test results), previous work has demonstrated that the sensitivity of the test is likely to lie somewhere between 50% to 100% with large variation depending on the estimation method used (Monaghan *et al.* (1994) [18] suggested a range of 68% to 95%, de la Rua-Domenech (2006) [19] a range between 75% and 95.5%, Clegg *et al.* (2011) [20] a range between 53% and 61% and Alvarez *et al.* (2012) [21] suggested a median sensitivity of 66–69% with high variability). The stage of infection of the animal tested and previous exposure to the SICCT have been shown to reduce the sensitivity of the test [22]. The consequences of the imperfect sensitivity of the SICCT test are not straightforward to assess due to the modality of the test which includes possible repeated testing of same individuals depending on the results of previous tests.

In this paper we propose a new methodology that can be used to incorporate imperfect test sensitivity into a discrete time survival modelling framework. We describe methods for conversion of the complex testing regime that exists for bTB in GB into an underlying discrete time (annual) response pattern of HBD responses for each herd. We then show the impact of the imperfect test on identified risk factors under a range of possible values for the sensitivity of the SICCT test.

## Materials and Methods

### Rationale

The aim of this paper was to investigate the impact of sensitivity of the single intra-dermal comparative cervical tuberculin (SICCT) test on the determination of risk factors associated with a positive bTB test result. As such, we did not attempt to infer or model the true disease or infection status of a cattle herd, but instead modelled the effect of having uncertain test results, irrespective of the underlying unknown infection status.

### Data Background

Since the discovery of a badger infected with bovine tuberculosis (bTB) in 1971, which raised questions on the role of badgers as a vector and reservoir of the pathogen, culling badgers became one possible measure to control the spread of the disease in addition to the control of cattle. The combination of the 1992 Badger Protection Act and the conflicting evidence regarding the efficacy of badger culling as a control measure for bTB, prompted a comprehensive review of the subject [23]. This resulted in 1998 in the design of a large scale clinical trial, the Randomised Badger Culling Trial (RBCT) [5]to evaluate the efficiency of two methods for culling badgers in altering the incidence of cattle herd breakdowns (HBDs) due to bTB. The RBCT ran from 1998 to 2005 (with the last surveys occurring in March 2006) and targeted regions of the UK with a high reported incidence of bTB. Ten ‘triplet’ areas were defined and following an initial survey of both farms and badgers’ social territories, each study area (triplet) was separated into a control trial area where no badger culling occurred and two treatment areas differing in the way badger culling was implemented. In the reactive treatment area, culling of badgers was performed on neighbouring farms where a bTB herd breakdown occurred. In the proactive treatment area, culling occurred over the whole area on an annual basis. For this paper, we restricted the analysis to one of the ten proactive culling trial areas (area B2). The area was chosen as it covers the full duration of the RBCT trial from 1998 to 2005. The B2 culling area comprised 174 herds corresponding to 167 distinct County Parish Holdings (or farms) registered to 164 registered land owners as per the RBCT database.

Multiple data sources were used to construct the response variable and the different predictors. Data related to herd bTB status and test results (including number of animals tested, number of reactors) were extracted from the Vetnet database. Animal movement data related to the farms present in the area were extracted from the British Cattle Movement Service (BCMS) Cattle Tracing System, and the number of animals moved aggregated by farm of origin and destination, and by calendar month. This was used to construct cattle movement predictors. From the data collected during the RBCT itself, we extracted information mostly relating to badgers. The RBCT database contained records of badger setts identified on different parcels of lands across the trial area. The badger data are also organised at a spatial level of badger social groups. The badger information can thus be related to the farm data using an association table relating badger social group, badger sett and registered land owner, these three variables forming a unique searching key between the badger trapping and survey data, and the farms. The final data source used was GIS information, which enabled building maps of both badger social groups and farmland, to define the neighbourhood structure for each farm (i.e. how many neighbours, each farm had) and to calculate distances between the centroid of each farm and the centre of the trial area. The GIS information was also used to identify farms with multiple parcels of land across the study area and to link each of these parcels to possible overlapping badger social groups.

### Response Variable – Relationship between Cattle Herd Breakdowns and Test Results

We chose as our response variable, a binary indicator of a cattle herd breakdown (HBD), considering both confirmed and unconfirmed HBD together as both have an impact on testing regime. By a HBD, we mean a herd being placed under restriction (unable to move animals off the farm) following a positive bTB test (which is the case for both confirmed and unconfirmed HBDs). Thus we modelled the risk of the herd being put under restriction and not the risk of becoming infected which is not known.

During the trial period, all cattle herds in area B2 were supposed to undergo a whole herd test on an annual basis. However, herds were not always tested each year with a total of 219 out of 1302 annual tests missing and 30 occurrences where a herd was not tested for two or more consecutive years. The reasons for the absence of a test were not always obvious from the data, and we considered such tests as “true missing tests”, i.e. a test was due and was not performed. This “missing test” could be due to the absence of eligible cattle in the herd, which could still leave a residual risk of bTB in the herd [15], or it may be an artefact of our time-interval selection, i.e. there may be just over 12 months between two consecutive tests.

### Discrete Time Survival Models

In discrete time survival modelling, we model the risk (or hazard) of an event happening, considering time as a succession of discrete time intervals during which the individual may or may not be at risk of the event occurring [4]. In our case, the event of interest was a bTB HBD. The unit of study was the herd, organised within farms within registered land owners; herds are uniquely identified with a County-Parish-Holding-Herd (CPHH) number. Observations were considered on an annual (i.e. 12 months) time interval to reflect the fact that herds followed an annual testing regime. Any shorter time step would result in a large amount of missing data because herds testing negative for bTB were rarely retested in the same year. Our annual time step (test-year) ran from the 1^st^ of February of each year to the 31^st^ of January of the following year, in what we called a “badger-year”. This choice was motivated by the trapping methodology used in the RBCT which was restricted to the 1^st^ of May through to the 31^st^ of January the following year to reduce the chance of trapping lactating badger sows with dependent cubs. By choosing to use a badger-year, we thus ensured (1) that any cub caught in a specific year was born in that year and (2) that two trapping seasons would not be considered within the same year. The change in temporal unit from calendar year to badger-year had no influence on the bTB testing regime in place for cattle.

Since some herds may have experienced multiple HBD events (on average a herd experienced 2.5 HBD during the study period), we included in the model a random effect at the individual occupier level assuming multiple herds associated with the same land owner will share the same random variation (there were only 10 more herds than owners). Based on results from previous work (Szmaragd *et al.* in preparation), we did not include a conditional autoregressive (CAR) spatial random effect as any spatial effects were assumed to be captured by the predictor variables. This resulted in a hierarchical structure with time periods nested within herds nested within owners but only owner level random effects:
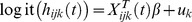


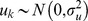

*h_ijk_*(*t*) is the hazard of an event occurring in time interval *t* during episode *i* for individual herd *j* for occupier *k.*

*X_ijk_*(*t*) are the predictor variables which might be time-varying or defined at the episode, individual herd, farm or occupier level. It also includes a polynomial function of the time at risk (see Table 1), which represents the baseline hazard of the event occurring based on the time since the previous event.

**Table 1:** List of the 12 possible patterns resulting from a 6-year pattern of herd status.

Pattern No	Pattern	Prior Probability	Response	No years since last event (years at risk)
1	100210	½ (1–*p*)^3^	100110	112311
2	101210	½ (1–*p*)^3^	101.10	112.11
3	100211	½ *p*(1–*p*)^2^	10011.	11231.
4	101211	½ *p*(1–*p*)^2^	101.1.	112.1.
5	100110	½ *p*(1–*p*)^2^	1001.0	1123.1
6	101110	½ *p*(1–*p*)^2^	101.0	112.1
7	100111	½ *p* ^2^(1–*p*)	1001.	1123.
8	101111	½ *p* ^2^(1–*p*)	101…	112…
9	111210	*p*(1–*p*)^2^	1…10	1…11
10	111211	*p* ^2^(1–*p*)	1…1.	1…1.
11	111110	*p* ^2^(1–*p*)	1….0	1….1
12	111111	*p* ^3^	1….	1….

The “.” indicates years where the herd was not at risk of a herd breakdown because it was already under restrictions. Those years are virtually ignored by the model fitting algorithm (Appendix S1).

*u_k_* is a random effect representing unobserved characteristics common to all episodes experienced by all herds sharing the same land owner *k.* We follow the common assumption that the *u_k_* are normally distributed with mean 0 and variance *σ_u_^2^.*

### Constructing the Response Variable in the Presence of Missing Test Results and Imperfect Test Sensitivity

When working with discrete time survival models (or event history data), the presence of missing outcomes for any individual impacts on the whole sequence of events/observations for that individual. Indeed, assuming the missing observation is an event (or respectively a non-event), this will alter (i) the probability of the following observation being an event and (ii) the time when the next episode starts.

**Figure 1:**
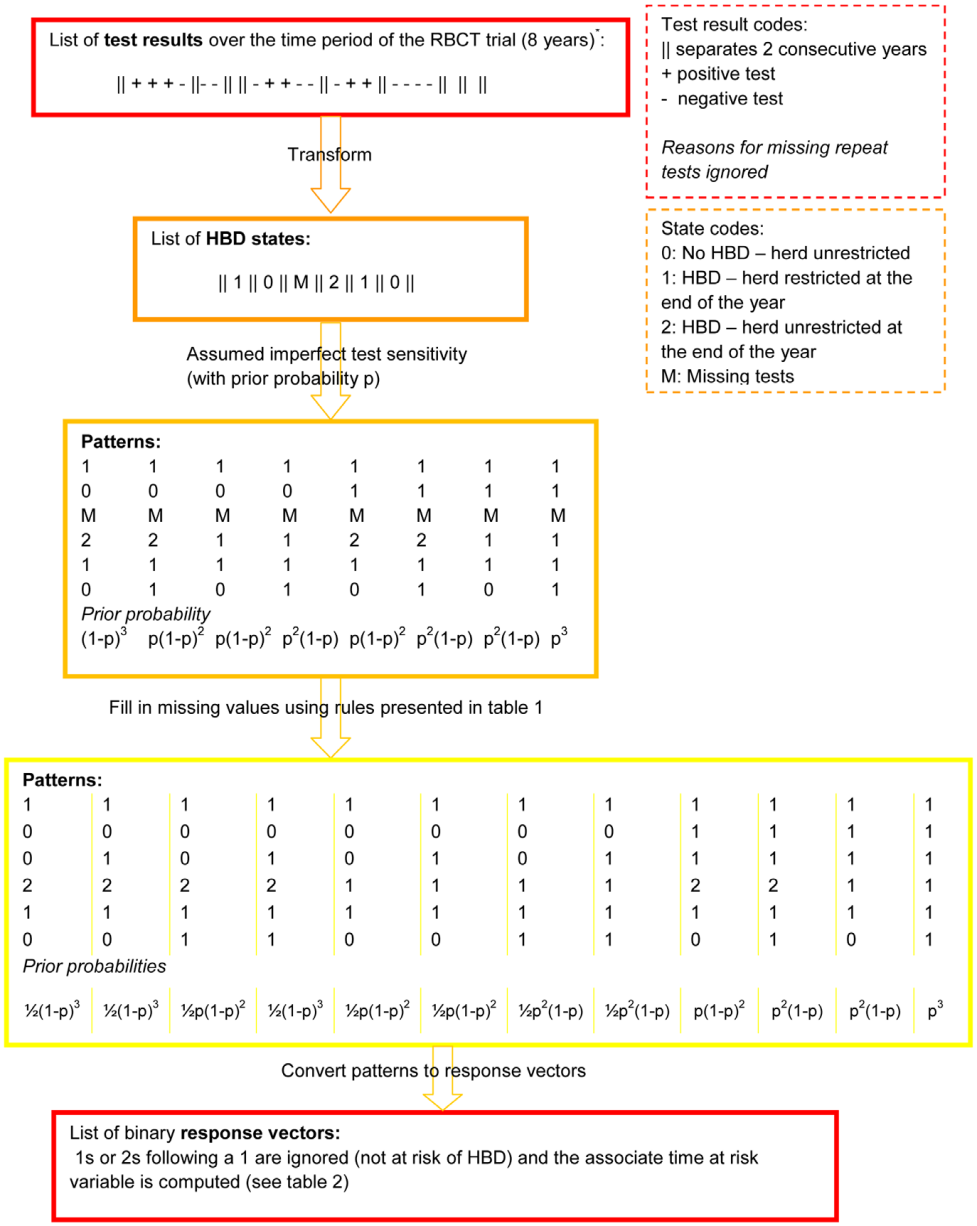
Diagram of the data preparation process. Example based on a hypothetical herd. *The herd might not exist for the whole duration of the study period. Missing year at the start and/or end of the study period are ignored, as the dataset does not to be balanced.

Before fitting the discrete time survival model described above, we transformed the observed responses based on the results of the tuberculin test into a set of patterns of HBD events which are the response variables used in the model and represent the underlying true disease status. The process used to create the pattern of HBD events is described in figure 1.

For each herd, our procedure starts with a list of test results grouped per badger-year. In the example of figure 1, this example herd had not been tested for years 3, 7 and 8. This list of test results is then transformed into a list (or vector) containing HBD states. We defined four states, which represents the restriction status of the herd based on the bTB test results:

**0** being defined as at least one bTB test occurred and all tests were negative, i.e. herd was not under-restriction.
**1** being defined as at least one HBD occurred and the herd was not unrestricted by the end of the period (2 consecutive negative tests in the period) by the end of the period, i.e. herd was still under-restriction at the period end.
**2** being defined as a HBD occurred but the herd was unrestricted by the end of the period (last 2 tests were negative in the period), i.e. herd was under-restriction for part of the period but the restrictions were lifted by the end of the period.
**M** no test occurred in this period and so the annual (test results) state is unknown.

The next step consists in accounting for the imperfect sensitivity of the test in defining the sequence of HBD. The imperfect sensitivity means that for each recorded 0 or 2 in the list of HBD based on the test results, there is a probability that in reality this state should actually be a 1 (i.e. the corresponding tuberculin test is truly positive). Thus for each 0 or 2 state in the list of HBD states for a particular herd, two possibilities arise: either the true state is actually a 1 (and the test was a false negative) with probability *p* or the test was a true negative and the true state is the same as the observed state with probability 1–*p*. This is propagated through the full sequence of HBD states for that herd leading to a set of possible patterns with associated prior probabilities depending on *p* (following a binomial probability distribution) as illustrated in figure 1.

The probability *p* is related to the test sensitivity but is actually closer to the negative predictive value and so will depend on the underlying prevalence of bTB in the population as explained below.

Some herds contained missing HBD states for certain years which are found through the set of patterns. We dealt with missing tests via a rule based approach so that some missing tests were filled in deterministically whilst others were uncertain and hence this resulted in additional possible response patterns being created for each herd. Our approach to filling in missing values is presented in Table 2 based on the herd states before and after the period with missing test(s). If the HBD states are identical before and after the period with missing test(s) and the before state is not a 2, then the missing HBD state take the same value. If the before state is either a 0 or 2 and the after state a 1 or 2, then the missing state can be either a 0 or 1, with equal probability. Finally if the before state is a 1 and the after state a 0, the missing state can be either 0, 1 or 2 with equal probability.

**Table 2:** The deterministic rules used for filling in missing test values.

After	0	1	2
Before			
0	0	0 or 1	0 or 1
1	0 or 1 or 2	1	1
2	0	0 or 1	0 or 1

The “Before” rows and “After” columns give the states of the herd in the years prior and after the missing test.

When the missing test values had been filled in and the patterns were fully defined (i.e. there are no longer any missing value), we transformed the resulting set of state vectors into response vectors indicating whether the herd was “at risk of HBD but had not broken down” ( = 0) or “was not at risk” because an event ( = 1) had occurred. If the herd was not at risk for two or more consecutive time steps (consecutive 1s or a 2 following a 1), the second and consecutive 1s were discarded from the data. We summarised in Table 1 the list of 12 patterns of HBD states represented in figure 1, with the corresponding response and time at risk vectors and the prior probability of each pattern being determined by the value of *p*.

Based on the fact that the test is designed with high specificity to avoid true negative herds being placed under restriction, we assumed for simplicity that the number of false positives was minimal and could be ignored. We expressed *p* as the ratio between the number of false negatives (FN) over the number of herds tested negative (FN + TN, TN being the number of true negative tests):
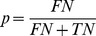



By introducing the number of true positive tests (TP) into the ratio, *p* was related to the sensitivity of the test:
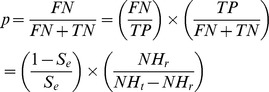
with *S_e_* being the sensitivity of the test and *NH_r_* and *NH_t_* being respectively the number of herds restricted (i.e. tested positive) and the number of herds tested. *NH_r_* and *NH_t_* were obtained from the Defra national statistics for TB for each year from 1998–2005 and for each county in the UK. As we are interested in areas within the RBCT, we compiled *NH_r_* and *NH_t_* as an overall average for the RBCT counties over the RBCT period. In addition to the perfect case of S_e_ = 1 or *p* = 0, we tested a range of sensitivities from 0.5 (50%) to 0.95 (95%) and these corresponded to values of *p* ranging from 0.008 to 0.153 (Table 3 provides the corresponding values of *p* for the sensitivities tested).

**Table 3:** Values of p used as prior probability of each pattern, for a range of possible sensitivity values.

Se	0.5	0.60	0.75	0.8	0.85	0.95
Prior probability value (p)*	0.153	0.102	0.0508	0.038	0.027	0.008

* Probability p = (NH_r_/(NH_t_-NH_r_))*((1–Se)/Se), with NH_r_ and NH_t_ being respectively the number of herds classified as reactors and the total number of herds. These numbers are overall averages computing from the statistics available from Defra for the British counties in the RBCT area between 1998 and 2005.

We considered the value for the sensitivity of the test to be a constant rather than a parameter in the model and populated the list of possible response patterns with associated prior probability distribution for each herd before running any statistical models, thus reducing the computation time required for estimating the model parameters. An extension that allows this parameter to be estimated is potentially feasible but would require a strong prior to ensure the model is identifiable and would require the pattern prior probabilities to be recalculated at each iteration (thus considerably increasing the computation time).

The true patterns (one of the set of the different possible patterns of true disease status for each herd) are treated as parameters to be updated as part of the Markov Chain Monte Carlo (MCMC) algorithm that we used to fit the model (see Appendix S1 for details).

MCMC algorithms are iterative simulation-based procedures, where parameters are grouped and updated in separate steps within each iteration. A pattern is therefore selected in one step of the algorithm in each iteration using the associated prior probability as a proposal distribution, and that pattern is then considered as known for the other steps in the iteration and the other model parameters (fixed effects estimates and variances) can be updated conditional on this fixed data.

We developed a general MCMC algorithm to fit discrete time survival models with potential multiple patterns for each herd, in the general case of assuming a 2-level random intercept model. We implemented our algorithm in special purpose computer code written in C++ (see Appendix S1 for the full description of the algorithm) and tested the code by comparing the results obtained by our algorithm with the ones given by the WinBUGS software package [24] for the cases with no missing tests.

Using the model fitting process described below, we estimated the best fitting models for each value of test sensitivity. We also considered the best fitting model found under the assumption of a perfect test (Se = 1 or *p* = 0), and looked at the effect on its parameter estimates of decreasing test sensitivity.

### Predictor Variables Considered

All the predictors were defined in relation to the response variable which was the risk of a HBD (binary variable) for a herd *j* in badger-year *y*. Some predictors were constant for the same herd across the different years, whilst some varied. Some predictors were also constructed by introducing a lag component, i.e. looking at the value of a predictor at a specific number of years prior to the year of the response (for simplicity the reference year will be termed “test-year” or badger-year).

We constructed nine badger variables: the number of badgers trapped in the test year and the previous year, the number of bTB positive badgers trapped in the test year and the previous year, the number of badgers estimated alive in the test year and the previous test year (constructed deterministically from dental records), the percentage of badgers infected in the test year and the previous test year and the cumulative number of badgers trapped from the start of culling to the test year. None of these predictors were significant when area B2 was considered in isolation so we omit further details.

The herd-level variables were herd enterprise (dairy-only, beef-only, mixed), mean herd size in test year and the previous year, number of calves born in the previous two years, new stock from homebred calves only (binary), whether the herd was depopulated in the 1–5 years or ever, before the test-year, number of cattle tested, number of reactors in the previous 1 to 5 years, cumulative number of reactors, missing test (in the test year or previous year), number of months since last test.

CPH/occupier level variables were the number of land parcels associated with the farm owner, average field size (ha); total farm area, stocking density, distance between the centroid of the farm and the centre of the trial area (m), number and average herd sizes of neighbouring herds, categorised by whether they were not tested, tested positive or negative for bTB.

Continuous variables were computed for each herd over a badger-year. If the herd tested negative in that year, the value of the variable was the average of the variable over the badger-year, while if the herd had a HBD, the value of the variable was obtained by averaging over the period from the start of the badger-year to the date of the first positive test or in the case of variables related to the number of reactors we used the sum over the whole badger-year.

The herd enterprise type was obtained from data records from both the RBCT database and historical records from VetNet for 2000 and 2002, allowing for some changes in herd enterprise type during the course of the trial.

There was a foot and mouth disease (FMD) epidemic in 2001 during which time some herds were depopulated. Herds were marked as depopulated if they decreased in size (through slaughter) by more than half in a month.

The set of predictors constructed from cattle movements were the number of cattle moved to a farm either directly or through market, categorised by year, test status of source farm (untested, positive, negative) in the previous and following year(s) and the testing frequency of the source farm. The number of cattle sold in the 12 or 12–24 months prior to the first unrestricted bTB test in any year were also considered as predictors. Rare movements (i.e. variables corresponding to types of movement where in total fewer than ten cattle were observed performing the type of movement in the whole dataset) were removed from the list of possible predictors prior to modelling.

### Model Fitting Process

**Figure 2:**
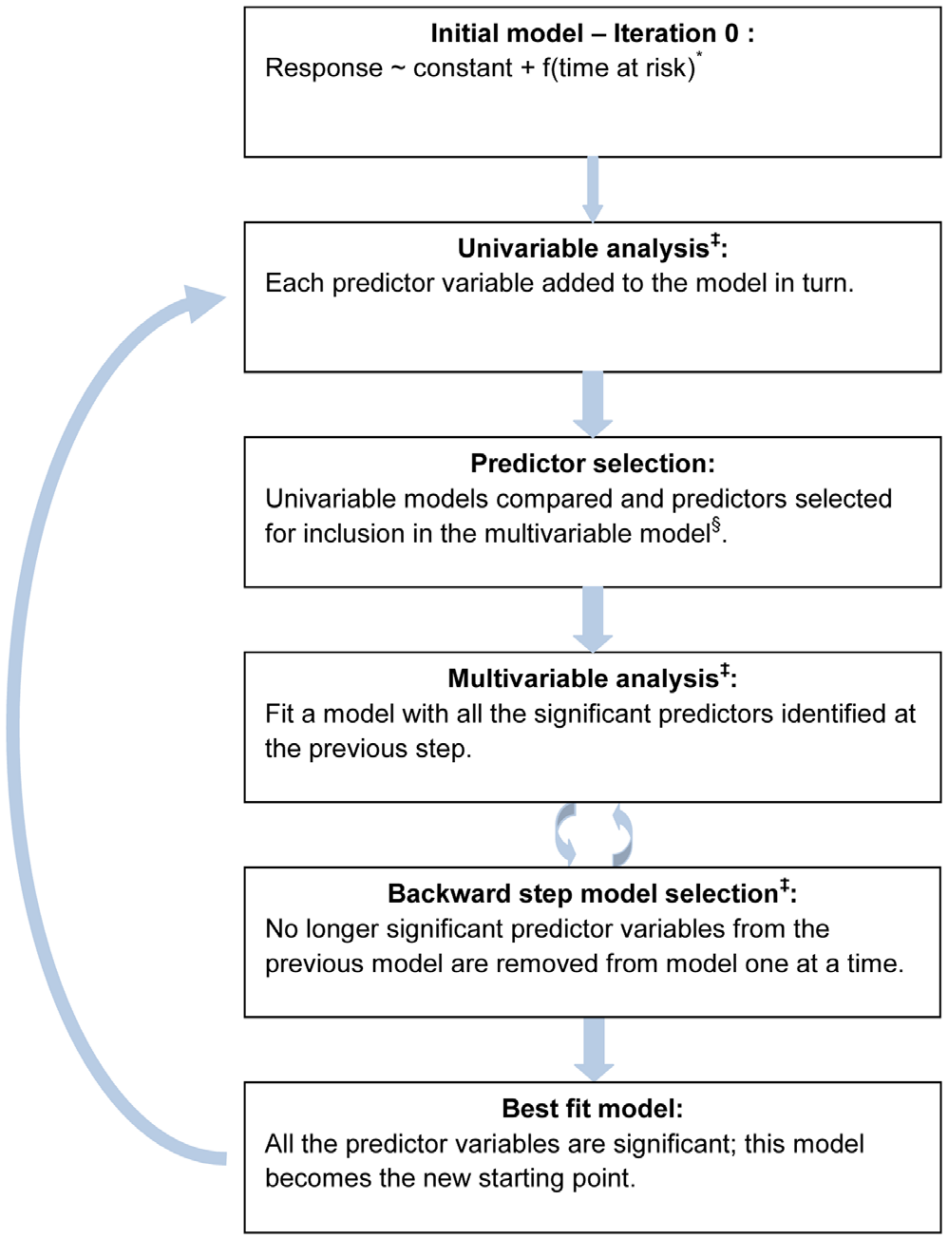
Diagram of the model fitting process. *cubic function of the time at risk variable was initially used. At the end of the first univariable iteration, the significance of each term will be assessed as part of the predictor selection step. None of the three terms were found significant and the time at risk variable was thus removed from the model ^§^A predictor is considered to be significant if its z-score (|posterior mean|/(posterior standard deviation)) is larger than 1.96 **^‡^**The models are fitted using the MCMC algorithm detailed in Appendix S1.

The model fitting process considered is similar to one given in Cox and Wermuth (1996) [25], and was performed in three main steps within an iterative loop (figure 2). The first step consisted of fitting univariable models where each predictor was added on its own to a base model containing random effects, the baseline hazard function and an intercept. When all predictors have been tested, they are ordered according to their “z-score” defined as the absolute value of the ratio of the posterior mean for the predictor to its posterior standard deviation. Predictors with a z-score larger than 1.96 are considered significant. In the second step, all the significant variables were included in a single model except for highly correlated variables (Spearman correlation coefficient more than 0.7). If two variables were strongly correlated then the variable with the higher z-score was preferentially included. The model including all these predictors was fitted and we then proceeded by removing no longer significant variables one at a time, refitting models as we went. This process continued until we reached a model where all the predictors were significant. In the third step, the optimum model for this round was used as the starting point for the next round. Step one was then repeated using this model as the base model. Once the remaining predictors had been tested in a univariable fashion, we checked for any new significant variable, which was not correlated with any predictor already in the model. We then repeated the second step, followed again by the third step (if necessary) until no more significant predictor could be added to the model.

Each model was run twice (to check for convergence to a unique mode) for 50,000 iterations following 5,000 burnin iterations. The initial values for the first chain were set to 0 for each predictor and to 0.1 for the variance of the random effects. The second chain was initialised using the opposite signed value of the coefficient values obtained for each predictor from the first chain, and using 1 for the variance of the random effects. Except for the choice of the pattern corresponding to the true disease status where we used the prior distribution derived from the test sensitivity, we did not have any prior information for the other parameters of the model. We, thus, used non-informative priors: uniform priors for fixed effects and a 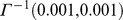 prior for the variance parameter.

## Results

Using data collected in one area of the Randomised Badger Control Trial (RBCT) where proactive culling of badgers occurred, we tested the effect of varying the bovine tuberculosis (bTB) test sensitivity from 50 to 100% on the identification of risk factors for bTB herd breakdown (HBD).

### Effect of Reduced Test Sensitivity on the Identification of bTB HBD Risk Factors

The best fitting model obtained under the assumption of perfect test sensitivity contained several predictors (see Table 4 for their full description), half of which were related to farm or herd demographic characteristics (including farm neighbourhood and the effect of the 2001 FMD epidemic); with the other half being related to cattle movements onto the farm (Table 5 **)**. This model highlighted that selling animals a year before being tested for bTB significantly reduced the risk of a HBD; this protective effect persisted across the range of sensitivity values. Perhaps not surprisingly, herds with larger number of animals tested and more calves born the year before the test had a significantly higher risk of a HBD. This increasing risk of HBD with larger herd size was also consistently found across the range of sensitivity albeit through different but highly correlated predictor variables such as the log of the mean herd size and number of calves born two years before the test (see Table S1 for the full correlation table). Previous HBD history as represented by the number of reactors two years before the test was also a significant risk factor which persisted across the range of sensitivities except when the test sensitivity dropped to 50% where it was replaced by the cumulative number of reactors in the previous four years (Spearman’s correlation coefficient of 0.58). The 2001-FMD outbreak appeared to mark an increase in the risk of HBD which persisted across the range of sensitivity values, however this could also be linked to a general increase in bTB prevalence with calendar time as has been reported across Great Britain.

**Table 4:** List of predictor variables appearing in the models.

Abbreviation	Full Variable name - Description	Statistics
LSoldPY	Log_e_(^#^animals sold, previous year)	1.71;1.84
LCalfPY	Log_e_(^#^Calves born, previous year)	2.61;1.86
LCalfPY2	Log_e_(^#^Calves born, two years previously)	2.30;1.94
LAT	Log_e_(^#^animals tested)	3.57;2.00
LAvHSY	Log_e_(mean herd size, in that year)	4.35;1.24
ReactPY2	^#^reactors found two years previously	0.56;2.74
CumReacPY4	Cumulative ^#^of reactors found in the previous four years	2.00;5.63
NeighPosY	^#^neighbour herds tested positive, same year	0.71;0.90
NeighPY	^#^neighbour herds, in the previous year	4.93;3.50
NeighNegPY	^#^neighbour herds tested negative, in the previous year	3.03;3.03
NeighPosPY	^#^neighbour herds tested positive, in the previous year	0.63;0.89
AvLHSNeighNegY	Mean log_e_(herd size of neighbour herds tested negative, same year)	0.61;1.14
AvHSNeighNotTPY	Mean(herd size of neighbour herds not tested, previous year)	14.22;36.45
FMD1	FMD Indicator 1; = 1 post February 2001	NA
FMD2	FMD Indicator 2; = 1 post February 2002	NA
FMD3	FMD Indicator 3; = Year indicator (1–3), before February 2001	NA
FMD4	FMD Indicator 4; = Year indicator (4–8), post February 2001	NA
BeefFarm	Beef-only enterprise (baseline category Dairy only)	57.8
MixedFarm	Mixed enterprise (baseline Dairy only)	30.7
Dep1	Depopulation indicator = 1 if herd depopulated in the past	51.7
LDirYSNegPast	Log_e_^#^animals bought directly in the test-year from a farm, which always tested negative for TBbefore the move	0.60;1.12
LDirPYSPosPY	Log_e_^#^animals bought directly in the previous test-year from a farm, which tested positive for TBin the 12 months before the move	0.03;0.23
DirYSNotTYRBCT	^#^animals bought directly in the test-year from a farm in the RBCT, which was not TB tested the12 months before the move	0.99;7.96
LMarkYSNotTFolY	Log_e_^#^animals bought through market in the test-year from a farm, which was nottested for TB the 12 months following the move	0.31;0.78
LMarkYSNegPYFq12	Log_e_^#^animals bought through market in the test-year from a farm in high TB risk area, which wastested negative for TB the 24–12 months before the move	0.07;0.33
LMarkYSNotTPYRBCT	Log_e_^#^animals bought through market in the test-year from a farm in the RBCT, which was nottested for TB the 24–12 months before the move	0.16;0.55
LMarkYSPosPY2RBCT	Log_e_^#^animals bought through market in the test-year from a farm in the RBCT, which was testedpositive for TB in the 36–24 months before the move	0.17;0.57
LDirPYSNegPast	Log_e_^#^animals bought directly in the previous test-year from a farm, which always tested negativefor TB before the move but after the 1^st^ of July 1996 when cattle passports were first implemented	0.08;0.35
LDirYSNegPYRBCT	Log_e_^#^animals bought directly in the test-year from a farm in the RBCT, which was tested negativefor TB the 24–12 months before the move	0.18;0.60
LDirYSPosPastRBCT	Log_e_^#^animals bought directly in the test-year from a farm in the RBCT, which was tested positivefor TB at some point before the move	0.47;0.99
LDirYSPosPYFq34	Log_e_^#^animals bought directly in the test-year from a farm in low TB risk area, which was testedpositive for TB the 24–12 months before the move	0.04;0.28
MarkYSPosFolY	^#^animals bought through market in the test-year from a farm, which was tested positive for TB the12 months following the move	2.20;11.97
MarkYSPosPY	^#^animals bought through market in the test-year from a farm, which was tested positive for TBthe 24–12 months before the move	7.23;43.14
MarkYSNegPY2	^#^animals bought through market in the test-year from a farm, which was tested negative for TBin the 36–24 months before the move	5.54;28.43
LMarkYSPosFolYFq34	Log_e_^#^animals bought through market in the test-year from a farm in low TB risk area, which wastested positive for TB the 12 months following the move	0.02;0.18
MarkYSNotTYFq34	^#^animals bought through market in the test-year from a farm in low TB risk area, which was nottested for TB the 12 months before the move	0.33;2.55

Table of abbreviations for the predictor variables found in the models, with full description and key statistics, being mean and standard deviation for continuous variables (expressed as per herd/per year) and frequencies for categorical variables over the number of herds present in the study area.

**Table 5:** Best fit models obtained for each sensitivity value tested.

	Se = 1	Se = 0.95	Se = 0.85	Se = 0.80	Se = 0.75	Se = 0.60	Se = 0.50
Parameters	Est (sd)	Est (sd)	Est (sd)	Est (sd)	Est (sd)	Est (sd)	Est (sd)
*Intercept*	*−5.13 (0.61)*	*−6.06 (0.60)*	*−4.83 (0.57)*	*−6.20 (0.66)*	*−6.56 (0.78)*	*−4.65 (0.65)*	*−5.26 (0.65)*
*LSoldPY*	*−0.97 (0.08)*	*−0.94 (0.07)*	*−0.92 (0.08)*	*−1.01 (0.08)*	*−1.04 (0.10)*	*−1.17 (0.11)*	*−1.20 (0.14)*
LCalfPY	0.58 (0.09)					0.79 (0.11)	
LCalfPY2		0.35 (0.07)	0.38 (0.07)	0.41 (0.08)	0.43 (0.09)		0.75 (0.14)
LAT	0.81 (0.12)		1.06 (0.13)			0.77 (0.14)	0.90 (0.15)
LAvHSY		1.12 (0.12)		1.25 (0.16)	1.31 (0.18)		
ReactPY2	0.12 (0.04)	0.10 (0.04)	0.09 (0.05)	0.10 (0.05)	0.11 (0.05)	0.15 (0.06)	
CumReacPY4							0.11(0.05)
NeighPosY	0.29 (0.11)	0.44 (0.10)	0.37 (0.12)	0.57 (0.12)	0.62 (0.13)	0.39 (0.15)	0.56 (0.19)
NeighPY							0.41 (0.11)
NeighNegPY				*−0.10 (0.05)*	*−0.12 (0.05)*		*−0.52 (0.13)*
NeighPosPY							*−0.66 (0.25)*
AvLHSNeighNegY	*−0.21 (0.08)*		*−0.19 (0.08)*			*−0.21 (0.10)*	
*AvHSNeighNotTPY*		*−0.01 (0.003)*		*−0.01 (0.003)*	*−0.012 (0.003)*		*−0.013 (0.005)*
FMD1						1.19 (0.28)	
FMD2	1.68 (0.29)	1.18 (0.25)	0.96 (0.24)	1.13 (0.27)	1.38 (0.28)	0.08 (0.01)	
FMD3	0.26 (0.12)						
FMD4							0.16 (0.05)
BeefFarm	**0.26 (0.30)**	0.15 (0.30)					
MixedFarm	0.66 (0.30)	0.64 (0.31)					
Dep1					1.34 (0.38)		
*LDirYSNegPast*	*−0.26 (0.11)*	*−0.32 (0.10)*		*−0.30 (0.12)*	*−0.39 (0.13)*		
*LDirPYSPosPY*	*−1.80 (0.79)*	*−1.73 (0.79)*	*−1.79 (0.79)*	*−1.88 (0.86)*	*−2.39 (0.94)*	*−2.16 (0.92)*	*−2.93 (1.18)*
*DirYSNotTYRBCT*	*−0.10 (0.05)*		*−0.10 (0.05)*			*−0.14 (0.07)*	
*LMarkYSNotTFolY*						*−1.10 (0.30)*	*−0.90 (0.33)*
*LMarkYSNegPYFq12*	*−0.91 (0.37)*		*−1.00 (0.38)*				
*LMarkYSNotTPYRBCT*		*−0.64 (0.28)*	*−0.66 (0.31)*	*−0.72 (0.31)*			
*LMarkYSPosPY2RBCT*						*−0.76 (0.37)*	
LDirPYSNegPast	0.81 (0.28)	0.80 (0.27)	0.90 (0.30)	0.92 (0.31)		0.99 (0.37)	1.33 (0.44)
LDirYSNegPYRBCT	0.40 (0.17)						
LDirYSPosPastRBCT						0.42 (0.15)	
LDirYSPosPYFq34	1.76 (0.60)	1.49 (0.46)	1.87 (0.61)	1.60 (0.50)	1.39 (0.55)		
MarkYSPosFolY	0.09 (0.02)	0.10 (0.02)	0.11 (0.03)	0.11 (0.02)			
MarkYSPosPY							0.06 (0.01)
MarkYSNegPY2					0.04 (0.01)		
LMarkYSPosFolYFq34	1.49 (0.65)						
MarkYSNotTYFq34						0.38 (0.13)	
Sigma^2^u	0.05 (0.08)	0.11 (0.13)	0.06 (0.10)	0.15 (0.17)	0.28 (0.36)	0.07 (0.11)	0.08 (0.16)

The values are given as posterior mean estimate plus posterior standard deviation (sd). The different predictors are grouped according to the type of predictor. The predictors in italic indicate protective factors and the predictors in bold indicate non-significant differences for categorical predictors.

With regard the effect of neighbouring cattle herds, herds with more neighbouring herds testing positive in the same year had a higher risk of HBD; while having larger neighbouring herds testing negative in the same year was protective against the risk of HBD; both effects probably reflect the testing regime. The increasing risk of HBD with more neighbouring herds testing positive persisted across the range of sensitivity values whereas the protective effect of being surrounded by large herds testing negative was replaced by the protective effect of having more neighbouring herds (or larger neighbouring) herds testing negative the previous year. While these different predictor variables are not highly correlated (supplementary Table S1), they represent a similar relationship between neighbouring herds and the probability of a HBD. When the sensitivity of the test dropped to 50%, two additional predictor variables became significant with opposite effects: the more neighbours a herd has in the previous year the higher the risk of a HBD (through higher probability of exposure and/or larger susceptible population) but the more neighbouring herds testing positive in the previous year the less the risk (possibly due to effective removal of the source of infection).

The type of herd enterprise (beef or mixed versus dairy) was a significant risk factor only under the assumption of perfect sensitivity or very high sensitivity (95%).

A number of variables related to purchase patterns were significant predictors for the risk of HBD in a given year but only two movements variables found under the assumption of perfect test sensitivity were consistent across the range of sensitivity values. These were the log of the number of animals bought the year before the test directly, from a farm which had tested positive at some point in the 12 months preceding the purchase, which was consistently associated with a decreased risk of HBD while the log number of animals bought the previous year from a farm which never tested positive before the move but after the 1^st^ of July 1996 when cattle passports were first implemented led to an increase in risk of HBD for all sensitivity values except for the best fiting model with 75% test sensitivity where it was replaced with the highly correlated indicator of depopulated herd. Two additional risk factors (including highly correlated factors), namely the number of animal bought through market from a farm testing positive in the year following the move and from a farm in a 3- or 4-yearly testing area which tested positive two years before the move, were found in the best fitting models for sensitivity value above 60%. The other movement predictor variables (a mixture of risk and protective factors) identified in the best fitting model in the case of the perfect sensitivity tended to disappear either partially (persisted for some sensitivity values but not others) or completely, while new movement predictors appeared.

### Effect of Reduced Test Sensitivity on the Parameter Estimates for bTB HBD Risk Factors

As the variables identified in the best fitting models for different values of test sensitivity varied, we investigated the effect of reducing test sensitivity on the parameter estimates obtained for the predictors of the best fitting model found under the assumption of perfect test sensitivity while keeping the model fixed. The rationale here was that epidemiological studies aiming at identifying risk factors from field data generally assume a perfect test (100% sensitivity). It is, however unclear what effect on the parameter estimates would be observed if this assumption was not met.

Our model fitting showed that while the size and the sign of the parameter estimates generally remained similar across the different sensitivity values, there was a consistent increase in the standard errors around these estimates with a decrease in sensitivity (Table 6). Some parameters ceased to be significant as sensitivity was reduced, mainly once this dropped below 60%, with the exception of one of the FMD indicators which lost significance as soon as the sensitivity of the test dropped below 95%. When the test sensitivity was less than 60%, the MCMC algorithm had convergence problems for the second chains with diffuse starting values. In our modelling approach, decreased test sensitivity led to more uncertainty in the actual pattern for each herd and the posterior distribution potentially became multi-modal. Here different patterns with similar prior probability of being correct will result in different predictors being significant. This also led to patterns with lower prior probability being more likely to be selected by the model than they were for higher sensitivity values.

**Table 6:** Parameter estimates by test sensitivities (Se), based on the best fit model under the assumption of a perfect test.

	Se = 1	Se = 0.95	Se = 0.85	Se = 0.80	Se = 0.75	Se = 0.60 *	Se = 0.50 *
Parameter	Est (sd)	Est (sd)	Est (sd)	Est (sd)	Est (sd)	Est (sd)	Est (sd)
*Intercept*	*−5.13 (0.61)*	*−5.21 (0.62)*	*−5.35 (0.67)*	*−5.39 (0.68)*	*−5.47 (0.67)*	*−5.40 (0.77)*	*−5.4 (0.79)*
*LSoldPY*	*−0.97 (0.08)*	*−0.99 (0.08)*	*−1.03 (0.09)*	*−1.05 (0.09)*	*−1.07 (0.09)*	*−1.14 (0.11)*	*−1.21 (0.12)*
LCalfPY	0.58 (0.09)	0.59 (0.09)	0.59 (0.1)	0.61 (0.1)	0.61 (0.1)	0.63 (0.11)	0.66 (0.13)
LAT	0.81 (0.12)	0.84 (0.12)	0.87 (0.13)	0.88 (0.14)	0.90 (0.13)	0.93 (0.15)	0.99 (0.16)
ReactPY2	0.12 (0.04)	0.13 (0.05)	0.13 (0.05)	0.13 (0.05)	0.14 (0.05)	0.15 (0.06)	0.17 (0.07)
NeighPosY	0.29 (0.11)	0.31 (0.12)	0.35 (0.13)	0.37 (0.13)	0.39 (0.14)	0.44 (0.16)	0.48 (0.17)
*AvLHSNeighNegY*	*-0.21 (0.08)*	*−0.21 (0.09)*	*−0.21 (0.9)*	*−0.21 (0.09)*	*−0.21 (0.09)*	***−0.22 (0.11)***	***−0.23 (0.12)***
FMD2	1.68 (0.29)	1.69 (0.29)	1.68 (0.30)	1.66 (0.31)	1.66 (0.31)	1.53 (0.35)	1.36 (0.38)
FMD3	0.26 (0.12)	**0.25 (0.13)**	**0.24 (0.14)**	**0.23 (0.14)**	**0.23 (0.15)**	**0.19 (0.17)**	**0.13 (0.19)**
BeefFarm	0.26 (0.3)	0.25 (0.3)	0.26 (0.32)	0.26 (0.32)	0.26 (0.33)	0.23 (0.36)	0.27 (0.4)
MixedFarm	0.66 (0.3)	0.66 (0.31)	0.66 (0.33)	0.66 (0.33)	0.68 (0.34)	0.67 (0.37)	0.72 (0.41)
*LDirYSNegPast*	*–0.26 (0.11)*	*−0.26 (0.12)*	*−0.26 (0.12)*	*−0.26 (0.13)*	*−0.27 (0.13)*	***−0.27 (0.15)***	***−0.27 (0.18)***
*LDirPYSPosPY*	*–1.8 (0.8)*	*−1.84 (0.82)*	*−1.90 (0.85)*	*−1.94 (0.86)*	*−1.98 (0.89)*	*−2.1 (0.95)*	*−2.24 (1.05)*
*DirYSNotTYRBCT*	*–0.1 (0.05)*	*−0.1 (0.05)*	*−0.11 (0.05)*	*−0.11 (0.05)*	*−0.12 (0.05)*	*−0.12 (0.06)*	***−0.12 (0.07)***
*LMarkYSNegPYFq12*	*−0.91 (0.37)*	*−0.98 (0.40)*	*−1.12 (0.48)*	*−1.21 (0.51)*	*−1.27 (0.54)*	*−1.55 (0.68)*	*−1.88 (0.82)*
LDirPYSNegPast	0.81 (0.28)	0.83 (0.29)	0.89 (0.3)	0.90 (0.31)	0.93 (0.32)	1.00 (0.36)	1.10 (0.42)
LDirYSNegPYRBCT	0.4 (0.17)	0.41 (0.18)	0.42 (0.19)	0.43 (0.19)	0.45 (0.2)	0.48 (0.23)	0.53 (0.26)
LDirYSPosPYFq34	1.76 (0.6)	1.85 (0.64)	2.02 (0.72)	2.11 (0.77)	2.21 (0.80)	2.33 (0.97)	**2.35 (1.23)**
MarkYSPosFolY	0.09 (0.02)	0.09 (0.02)	0.1 (0.02)	0.10 (0.02)	0.10 (0.02)	0.11 (0.03)	0.11 (0.03)
LMarkYSPosFolYFq34	1.49 (0.65)	1.64 (0.81)	**1.97 (1.04)**	**2.1 (1.11)**	**2.41 (2.18)**	**187 (294)**	**3.5E^8^ (5E^8^)**
Sigma^2^u	0.05 (0.08)	0.05 (0.08)	0.05 (0.08)	0.05 (0.09)	0.05 (0.09)	0.04 (0.08)	0.06 (0.09)

Posterior mean estimates (posterior standard deviations) obtained by running 2 independent MCMC chains of 50,000 iterations after 5,000 burnin. In italic are highlighted predictors which have a protective effect. Non significant estimates are highlighted in bold.

* Problem with convergence of the two chains encountered.

## Discussion

In this paper, we have presented a new approach for fitting discrete time survival models where the response variable contains missing values and at the same time where the known values of the response variable are surrounded by a certain amount of uncertainty. This type of data is especially found in epidemiology where the response variable relates to disease status, which itself is dependent on a (possibly imperfect) diagnostic test. The presence of missing values alone leads to a response variable which is not uniquely defined but instead takes the form of a set of multiple possible outcomes. This set of potential outcomes is further increased by the uncertainty relating to the imperfect nature of the test used, which affects the prior probability of each response pattern.

We developed a Bayesian model to deal with such cases and applied it to historical data on herd breakdown (HBD) with bovine tuberculosis (bTB). We found that accounting for the imperfect sensitivity of the diagnostic test affects which risk factors are significantly associated with a bTB herd breakdown and in particular that a decreased test sensitivity leads to larger confidence intervals around the parameter estimates of each risk factors.

A small set of predictors (mostly non cattle-purchase variables) were consistently significant across the best fitting models for each different value of test sensitivity. The cattle-purchase variables were the most affected by decreasing sensitivity values with only two out of the nine predictors identified for the perfect sensitivity best fitting model being significant across the whole range of sensitivity values. Whatever the true sensitivity of the test, larger herds (as represented by higher number of calves born, larger herd sizes, or greater number of animals tested) and herds with a history of bTB and larger number reactors found at a HBD 2 years previous to the current test had an increased risk of HBD while selling an animal before the test was protective. The marked increase in HBD after the 2001-FMD outbreak was also unaffected by decreased sensitivity. Our analyses also confirmed similarity in the pattern of HBD between neighbouring herds, which held through the range of sensitivity values with some variations in the predictors. As predictor variables we only considered the number of neighbouring herds that tested positive or negative rather than the number of neighbouring herds that were actually positive or negative. A possible extension of our modelling approach, when assuming less than perfect test sensitivity, would be to recalculate numbers of neighbours that are truly positive/negative for a given herd, at each iteration of the algorithm, for use as predictors but we will consider this extension elsewhere.

The data we analysed were collected as part of a large scale clinical trial/field experiment designed to detect gross overall effects between different treatments (culling and survey) in an area. It was therefore, as such, not intended for the fine grain analyses we have performed. We included cattle movement predictors alongside badger and herd-demographic predictors. This resulted in a large number of predictors to be considered as possible risk factors, which can lead to the usual problems of multiple comparisons and might explain why some of the effects found were perhaps counter intuitive. However, the aim of this paper was to investigate the effect of decreased test sensitivity on the risk factors determined rather than identify these risk factors *per-se* Under decreasing values of sensitivity, slightly different sets of predictors appeared in the best fitting model, but with some of the predictors considered across models being strongly correlated with one another (see Table S1). Our statistical modelling approach identifies associations between predictor variables and HBD rather than causations and so care has to be taken in acting upon the findings, especially given the large number of predictors considered.

Our analysis of the effect of reduced bTB test sensitivity highlighted an increase in the uncertainty surrounding the parameter estimates identified by the models, with some predictors losing significance altogether. This has important consequences for epidemiological field studies aiming at identifying risk factors for infectious disease, as they are based on the assumption of perfect test sensitivity. Given that the published estimates of sensitivity are around 60%–75%, our analysis suggests this could explain the difficulty in finding a consistent and reliable set of risk factors for bTB. It is therefore essential that estimates of bTB test sensitivity (at individual and herd level) be confirmed in the field, possibly by using complementary test diagnostics and analyses adjusted for the lack of sensitivity [20], [21]. Increasing sample sizes to account for imperfect test sensitivity is already advised when designing cross-sectional studies, but we have shown that the same holds true for longitudinal data. Future longitudinal field studies would benefit from being sufficiently large by adjusting for a less than perfect test sensitivity when calculating the sample sizes, if reliable conclusions are to be drawn from the data.

The method we presented here is not just limited to the study of bTB risk factors or to epidemiological data in general. It could be applied to a wide range of datasets where the response variable may be surrounded by some level of uncertainty, which could influence the associated predictors. The deterministic set of rules used to resolve the case of missing data was specific to this dataset, being based on the testing regime which produces the response variables, but could be adapted to other scenarios. The solution we proposed illustrates how one could implement a similar approach when confronted with missing data in discrete time survival analysis, instead of removing the observations corresponding to a subject with missing responses. In our case, the data only had a simple non-spatial two level random effects structure but our method can be extended to include spatial error structure or higher levels of data structure. The only drawback of such methods is that they are computationally intensive and therefore slow to run.

## Supporting Information

Table S1 **Full correlation tables for the predictor variables appearing in** **table 5** **of the main text.** Only Spearman or Pearson correlation coefficients over 0.20 are shown. In bold, are indicated Spearman correlations over 0.5.
(DOCX)

Appendix S1 **MCMC algorithm.** Expression of the likelihood for discrete time survival model with multiple patterns.
(DOCX)
