# Supplementary material for: Impact of Imperfect Test Sensitivity on Determining Risk Factors: The Case of Bovine Tuberculosis
Source: PLoS One. 2012 Aug 13;7(8):e43116. doi: 10.1371/journal.pone.0043116 (PMC3418251; doi:10.1371/journal.pone.0043116)
Supplement: Table S1 — Full correlation tables for the predictor variables appearing in table 5 of the main text. Only Spearman or Pearson correlation coefficients over 0.20 are shown. In bold, are indicated Spearman correlations over 0.5. (DOCX) [file pone.0043116.s001.docx]

**Table S1: Full correlation tables for the predictor variables appearing in table 5 of the main text**. Only Spearman or Pearson correlation coefficient over 0.20 are shown. In bold, are indicated Spearman correlations over 0.5.

| **Variable 1** | **Variable 2** | **Spearman** | **Pearson** |
| --- | --- | --- | --- |
| Mean log_e_(herd size of neighbour herds tested negative, same year) | FMD Indicator 3; = Year indicator (1-3), before February 2001 | -0.42867 | -0.48192 |
| # neighbour herds tested positive, same year | Mean log_e_(herd size of neighbour herds tested negative, same year) | -0.36519 | -0.28659 |
| Log_e_(# animals tested) | FMD Indicator 3; = Year indicator (1-3), before February 2001 | -0.35889 | -0.45702 |
| Log_e_(# Calves born, previous year) | Beef-only enterprise (baseline category Dairy only) | -0.34485 | -0.33497 |
| # neighbour herds tested negative, in the previous year | FMD Indicator 4; = Year indicator (4-8), post February 2001 | -0.31596 | -0.24152 |
| FMD Indicator 2; = 1 post February 2002 | FMD Indicator 3; = Year indicator (1-3), before February 2001 | -0.2987 | -0.2987 |
| Log_e_(mean herd size, in that year) | Beef-only enterprise (baseline category Dairy only) | -0.25082 | -0.20544 |
| Log_e_(# Calves born, two years previously) | Beef-only enterprise (baseline category Dairy only) | -0.24753 | -0.24402 |
| Depopulation indicator = 1 if herd depopulated in the past | Log_e_ # animals bought directly in the test-year from a farm, which always tested negative for TB before the move) | -0.2196 | -0.17748 |
| FMD Indicator 2; = 1 post February 2002 | Mixed enterprise (baseline Dairy only) | -0.20668 | -0.20668 |
| Log_e_ # animals bought through market in the test-year from a farm, which was not tested for TB the 12 months following the move | Log_e_ # animals bought through market in the test-year from a farm in low TB risk area, which was tested positive for TB the 12 months following the move | 0.20155 | 0.235554 |
| Log_e_(# Calves born, previous year) | # reactors found two years previously | 0.202031 | 0.162573 |
| # animals bought through market in the test-year from a farm, which was tested negative for TB in the 36-24 months before the move | Log_e_ # animals bought through market in the test-year from a farm in low TB risk area, which was tested positive for TB the 12 months following the move | 0.202666 | 0.234246 |
| FMD Indicator 3; = Year indicator (1-3), before February 2001 | Depopulation indicator = 1 if herd depopulated in the past | 0.203126 | 0.203126 |
| FMD Indicator 2; = 1 post February 2002 | Beef-only enterprise (baseline category Dairy only) | 0.203616 | 0.203616 |
| Log_e_ # animals bought through market in the test-year from a farm in the RBCT, which was not tested for TB the 24-12 months before the move | Log_e_ # animals bought directly in the test-year from a farm in the RBCT, which was tested positive for TB at some point before the move | 0.20437 | 0.207274 |
| Log_e_(# Calves born, two years previously) | # reactors found two years previously | 0.204991 | 0.174455 |
| Log_e_(# Calves born, two years previously) | Mean(herd size of neighbour herds not tested, previous year) | 0.205751 | 0.06708 |
| # animals bought directly in the test-year from a farm in the RBCT, which was not TB tested the 12 months before the move | Log_e_ # animals bought directly in the test-year from a farm in low TB risk area, which was tested positive for TB the 24-12 months before the move | 0.206291 | 0.050126 |
| Cumulative # of reactors found in the previous four years | # neighbour herds, in the previous year | 0.207031 | 0.355917 |
| Log_e_(# animals sold, previous year) | FMD Indicator 3; = Year indicator (1-3), before February 2001 | 0.208044 | 0.199967 |
| Log_e_(# Calves born, two years previously) | # neighbour herds tested positive, in the previous year | 0.208907 | 0.194883 |
| Log_e_ # animals bought directly in the test-year from a farm, which always tested negative for TB before the move) | Log_e_ # animals bought through market in the test-year from a farm in the RBCT, which was tested positive for TB in the 36-24 months before the move | 0.210073 | 0.215606 |
| Log_e_ # animals bought through market in the test-year from a farm, which was not tested for TB the 12 months following the move | Log_e_ # animals bought directly in the test-year from a farm in the RBCT, which was tested positive for TB at some point before the move | 0.210802 | 0.217191 |
| # animals bought directly in the test-year from a farm in the RBCT, which was not TB tested the 12 months before the move | Log_e_ # animals bought directly in the test-year from a farm in the RBCT, which was tested negative for TB the 24-12 months before the move | 0.211234 | 0.300547 |
| Mean log_e_(herd size of neighbour herds tested negative, same year) | FMD Indicator 2; = 1 post February 2002 | 0.213285 | 0.21127 |
| Log_e_ # animals bought directly in the test-year from a farm, which always tested negative for TB before the move) | # animals bought through market in the test-year from a farm in low TB risk area, which was not tested for TB the 12 months before the move | 0.214094 | 0.146693 |
| # neighbour herds tested positive, same year | # neighbour herds tested positive, in the previous year | 0.214698 | 0.238703 |
| Log_e_ # animals bought through market in the test-year from a farm, which was not tested for TB the 12 months following the move | Log_e_ # animals bought directly in the test-year from a farm in low TB risk area, which was tested positive for TB the 24-12 months before the move | 0.217277 | 0.182117 |
| Log_e_ # animals bought directly in the test-year from a farm in the RBCT, which was tested positive for TB at some point before the move | # animals bought through market in the test-year from a farm, which was tested negative for TB in the 36-24 months before the move | 0.221916 | 0.191928 |
| Log_e_(# Calves born, previous year) | Mixed enterprise (baseline Dairy only) | 0.22387 | 0.205751 |
| Log_e_ # animals bought directly in the test-year from a farm, which always tested negative for TB before the move) | # animals bought through market in the test-year from a farm, which was tested positive for TB the 12 months following the move | 0.225223 | 0.198812 |
| # animals bought through market in the test-year from a farm, which was tested positive for TB the 24-12 months before the move | Log_e_ # animals bought through market in the test-year from a farm in low TB risk area, which was tested positive for TB the 12 months following the move | 0.226286 | 0.167178 |
| # animals bought directly in the test-year from a farm in the RBCT, which was not TB tested the 12 months before the move | Log_e_ # animals bought through market in the test-year from a farm in the RBCT, which was not tested for TB the 24-12 months before the move | 0.230568 | 0.119619 |
| Log_e_(# animals sold, previous year) | Mean(herd size of neighbour herds not tested, previous year) | 0.230591 | 0.147477 |
| Log_e_(# Calves born, previous year) | # neighbour herds tested negative, in the previous year | 0.230678 | 0.276135 |
| Log_e_ # animals bought through market in the test-year from a farm in the RBCT, which was tested positive for TB in the 36-24 months before the move | Log_e_ # animals bought directly in the test-year from a farm in the RBCT, which was tested positive for TB at some point before the move | 0.233217 | 0.223392 |
| Log_e_ # animals bought directly in the test-year from a farm, which always tested negative for TB before the move) | Log_e_ # animals bought through market in the test-year from a farm in the RBCT, which was not tested for TB the 24-12 months before the move | 0.233328 | 0.228458 |
| Log_e_(mean herd size, in that year) | # reactors found two years previously | 0.235931 | 0.177626 |
| Log_e_ # animals bought directly in the test-year from a farm in the RBCT, which was tested positive for TB at some point before the move | # animals bought through market in the test-year from a farm, which was tested positive for TB the 12 months following the move | 0.236062 | 0.185929 |
| Log_e_(mean herd size, in that year) | # neighbour herds tested negative, in the previous year | 0.236071 | 0.278768 |
| Log_e_ # animals bought directly in the test-year from a farm, which always tested negative for TB before the move) | # animals bought through market in the test-year from a farm, which was tested positive for TB the 24-12 months before the move | 0.243022 | 0.216965 |
| Log_e_(# animals tested) | # neighbour herds, in the previous year | 0.245808 | 0.174501 |
| Log_e_(# Calves born, two years previously) | # neighbour herds, in the previous year | 0.247263 | 0.291671 |
| Log_e_ # animals bought directly in the test-year from a farm, which always tested negative for TB before the move) | Log_e_ # animals bought directly in the test-year from a farm in the RBCT, which was tested negative for TB the 24-12 months before the move | 0.250651 | 0.234971 |
| Log_e_ # animals bought directly in the test-year from a farm, which always tested negative for TB before the move) | Log_e_ # animals bought directly in the test-year from a farm in the RBCT, which was tested positive for TB at some point before the move | 0.252442 | 0.256906 |
| Log_e_ # animals bought directly in the test-year from a farm, which always tested negative for TB before the move) | Log_e_ # animals bought directly in the test-year from a farm in low TB risk area, which was tested positive for TB the 24-12 months before the move | 0.252968 | 0.297522 |
| Log_e_ # animals bought directly in the previous test-year from a farm, which tested positive for TB in the 12 months before the move | Log_e_ # animals bought directly in the previous test-year from a farm, which always tested negative for TB before the move | 0.262665 | 0.227213 |
| Log_e_ # animals bought directly in the test-year from a farm in the RBCT, which was tested positive for TB at some point before the move | # animals bought through market in the test-year from a farm, which was tested positive for TB the 24-12 months before the move | 0.263305 | 0.180759 |
| # neighbour herds tested negative, in the previous year | # neighbour herds tested positive, in the previous year | 0.267435 | 0.271979 |
| Log_e_(# animals sold, previous year) | Log_e_(mean herd size, in that year) | 0.269032 | 0.299514 |
| # animals bought through market in the test-year from a farm, which was tested positive for TB the 12 months following the move | Log_e_ # animals bought through market in the test-year from a farm in low TB risk area, which was tested positive for TB the 12 months following the move | 0.271368 | 0.22398 |
| Log_e_(# Calves born, previous year) | # neighbour herds, in the previous year | 0.273881 | 0.316945 |
| FMD Indicator 2; = 1 post February 2002 | # animals bought through market in the test-year from a farm, which was tested positive for TB the 24-12 months before the move | 0.276139 | 0.138492 |
| Log_e_ # animals bought through market in the test-year from a farm in the RBCT, which was tested positive for TB in the 36-24 months before the move | # animals bought through market in the test-year from a farm in low TB risk area, which was not tested for TB the 12 months before the move | 0.27623 | 0.234247 |
| Log_e_ # animals bought through market in the test-year from a farm in high TB risk area, which was tested negative for TB the 24-12 months before the move | # animals bought through market in the test-year from a farm, which was tested negative for TB in the 36-24 months before the move | 0.276422 | 0.019762 |
| Log_e_(# animals sold, previous year) | Log_e_(# Calves born, previous year) | 0.281233 | 0.293558 |
| FMD Indicator 2; = 1 post February 2002 | # animals bought through market in the test-year from a farm, which was tested positive for TB the 12 months following the move | 0.282457 | 0.191642 |
| Log_e_(# animals sold, previous year) | FMD Indicator 2; = 1 post February 2002 | 0.287599 | 0.29714 |
| Log_e_(# animals tested) | Cumulative # of reactors found in the previous four years | 0.300275 | 0.190975 |
| Log_e_ # animals bought through market in the test-year from a farm in the RBCT, which was not tested for TB the 24-12 months before the move | # animals bought through market in the test-year from a farm in low TB risk area, which was not tested for TB the 12 months before the move | 0.303257 | 0.295186 |
| Log_e_(# Calves born, previous year) | Cumulative # of reactors found in the previous four years | 0.306156 | 0.258261 |
| Log_e_ # animals bought directly in the test-year from a farm, which always tested negative for TB before the move) | # animals bought through market in the test-year from a farm, which was tested negative for TB in the 36-24 months before the move | 0.30819 | 0.240706 |
| Log_e_(# Calves born, two years previously) | Cumulative # of reactors found in the previous four years | 0.31019 | 0.269389 |
| Log_e_ # animals bought through market in the test-year from a farm in low TB risk area, which was tested positive for TB the 12 months following the move | # animals bought through market in the test-year from a farm in low TB risk area, which was not tested for TB the 12 months before the move | 0.314052 | 0.621721 |
| # animals bought through market in the test-year from a farm, which was tested positive for TB the 24-12 months before the move | # animals bought through market in the test-year from a farm in low TB risk area, which was not tested for TB the 12 months before the move | 0.319618 | 0.288906 |
| FMD Indicator 1; = 1 post February 2001 | FMD Indicator 4; = Year indicator (4-8), post February 2001 | 0.326784 | 0.326784 |
| # animals bought through market in the test-year from a farm, which was tested positive for TB the 12 months following the move | # animals bought through market in the test-year from a farm in low TB risk area, which was not tested for TB the 12 months before the move | 0.328611 | 0.277401 |
| Log_e_ # animals bought directly in the test-year from a farm, which always tested negative for TB before the move) | Log_e_ # animals bought through market in the test-year from a farm, which was not tested for TB the 12 months following the move | 0.331609 | 0.353884 |
| FMD Indicator 1; = 1 post February 2001 | FMD Indicator 3; = Year indicator (1-3), before February 2001 | 0.337283 | 0.337283 |
| Log_e_ # animals bought directly in the test-year from a farm, which always tested negative for TB before the move) | # animals bought directly in the test-year from a farm in the RBCT, which was not TB tested the 12 months before the move | 0.343756 | 0.245885 |
| Log_e_(mean herd size, in that year) | # neighbour herds, in the previous year | 0.349639 | 0.361203 |
| # neighbour herds tested positive, same year | # neighbour herds, in the previous year | 0.350274 | 0.384518 |
| # neighbour herds, in the previous year | # neighbour herds tested positive, in the previous year | 0.352194 | 0.372005 |
| # animals bought through market in the test-year from a farm, which was tested negative for TB in the 36-24 months before the move | # animals bought through market in the test-year from a farm in low TB risk area, which was not tested for TB the 12 months before the move | 0.356191 | 0.371627 |
| Mean(herd size of neighbour herds not tested, previous year) | FMD Indicator 2; = 1 post February 2002 | 0.356781 | 0.35839 |
| Mean(herd size of neighbour herds not tested, previous year) | FMD Indicator 1; = 1 post February 2001 | 0.361323 | 0.310484 |
| Log_e_ # animals bought directly in the test-year from a farm in the RBCT, which was tested negative for TB the 24-12 months before the move | Log_e_ # animals bought directly in the test-year from a farm in the RBCT, which was tested positive for TB at some point before the move | 0.362139 | 0.376781 |
| Log_e_(mean herd size, in that year) | Cumulative # of reactors found in the previous four years | 0.374252 | 0.300434 |
| Log_e_(# Calves born, two years previously) | # neighbour herds tested negative, in the previous year | 0.379795 | 0.388902 |
| # animals bought directly in the test-year from a farm in the RBCT, which was not TB tested the 12 months before the move | Log_e_ # animals bought directly in the test-year from a farm in the RBCT, which was tested positive for TB at some point before the move | 0.38397 | 0.347195 |
| FMD Indicator 2; = 1 post February 2002 | FMD Indicator 4; = Year indicator (4-8), post February 2001 | 0.409667 | 0.409667 |
| Log_e_(# animals sold, previous year) | FMD Indicator 1; = 1 post February 2001 | 0.415167 | 0.419474 |
| Log_e_ # animals bought through market in the test-year from a farm, which was not tested for TB the 12 months following the move | # animals bought through market in the test-year from a farm in low TB risk area, which was not tested for TB the 12 months before the move | 0.428338 | 0.406388 |
| Log_e_(# animals sold, previous year) | Log_e_(# Calves born, two years previously) | 0.428478 | 0.442412 |
| Depopulation indicator = 1 if herd depopulated in the past | Log_e_ # animals bought directly in the previous test-year from a farm, which tested positive for TB in the 12 months before the move | 0.433329 | 0.386802 |
| Log_e_(# Calves born, two years previously) | Log_e_(# animals tested) | 0.471872 | 0.341257 |
| Log_e_ # animals bought through market in the test-year from a farm, which was not tested for TB the 12 months following the move | Log_e_ # animals bought through market in the test-year from a farm in the RBCT, which was tested positive for TB in the 36-24 months before the move | 0.495708 | 0.570925 |
| Log_e_ # animals bought through market in the test-year from a farm in the RBCT, which was not tested for TB the 24-12 months before the move | Log_e_ # animals bought through market in the test-year from a farm in the RBCT, which was tested positive for TB in the 36-24 months before the move | 0.496404 | 0.589982 |
| **Log_e_ # animals bought through market in the test-year from a farm in the RBCT, which was tested positive for TB in the 36-24 months before the move** | **# animals bought through market in the test-year from a farm, which was tested negative for TB in the 36-24 months before the move** | **0.519437** | **0.613385** |
| **Log_e_ # animals bought through market in the test-year from a farm, which was not tested for TB the 12 months following the move** | **Log_e_ # animals bought through market in the test-year from a farm in the RBCT, which was not tested for TB the 24-12 months before the move** | **0.540968** | **0.608202** |
| **Log_e_ # animals bought through market in the test-year from a farm, which was not tested for TB the 12 months following the move** | **# animals bought through market in the test-year from a farm, which was tested positive for TB the 12 months following the move** | **0.54457** | **0.507735** |
| **Log_e_ # animals bought through market in the test-year from a farm in the RBCT, which was not tested for TB the 24-12 months before the move** | **# animals bought through market in the test-year from a farm, which was tested positive for TB the 24-12 months before the move** | **0.556885** | **0.52948** |
| **Mean(herd size of neighbour herds not tested, previous year)** | **FMD Indicator 4; = Year indicator (4-8), post February 2001** | **0.558767** | **0.711402** |
| **# reactors found two years previously** | **Cumulative # of reactors found in the previous four years** | **0.583607** | **0.672607** |
| **Log_e_ # animals bought through market in the test-year from a farm in the RBCT, which was not tested for TB the 24-12 months before the move** | **# animals bought through market in the test-year from a farm, which was tested positive for TB the 12 months following the move** | **0.594862** | **0.611769** |
| **Log_e_(# Calves born, previous year)** | **Log_e_(# animals tested)** | **0.600241** | **0.448026** |
| **Log_e_ # animals bought through market in the test-year from a farm in the RBCT, which was not tested for TB the 24-12 months before the move** | **# animals bought through market in the test-year from a farm, which was tested negative for TB in the 36-24 months before the move** | **0.601445** | **0.629461** |
| **Log_e_ # animals bought through market in the test-year from a farm in the RBCT, which was tested positive for TB in the 36-24 months before the move** | **# animals bought through market in the test-year from a farm, which was tested positive for TB the 12 months following the move** | **0.61565** | **0.63316** |
| **Log_e_ # animals bought through market in the test-year from a farm, which was not tested for TB the 12 months following the move** | **# animals bought through market in the test-year from a farm, which was tested positive for TB the 24-12 months before the move** | **0.619617** | **0.52171** |
| **# neighbour herds, in the previous year** | **# neighbour herds tested negative, in the previous year** | **0.633692** | **0.778875** |
| **Log_e_(# Calves born, two years previously)** | **Log_e_(mean herd size, in that year)** | **0.637628** | **0.595006** |
| **Log_e_ # animals bought through market in the test-year from a farm in the RBCT, which was tested positive for TB in the 36-24 months before the move** | **# animals bought through market in the test-year from a farm, which was tested positive for TB the 24-12 months before the move** | **0.654697** | **0.604821** |
| **Log_e_ # animals bought through market in the test-year from a farm, which was not tested for TB the 12 months following the move** | **# animals bought through market in the test-year from a farm, which was tested negative for TB in the 36-24 months before the move** | **0.710563** | **0.580169** |
| **Log_e_(# animals tested)** | **Log_e_(mean herd size, in that year)** | **0.723111** | **0.554822** |
| **# animals bought through market in the test-year from a farm, which was tested positive for TB the 12 months following the move** | **# animals bought through market in the test-year from a farm, which was tested negative for TB in the 36-24 months before the move** | **0.725849** | **0.937446** |
| **# animals bought through market in the test-year from a farm, which was tested positive for TB the 12 months following the move** | **# animals bought through market in the test-year from a farm, which was tested positive for TB the 24-12 months before the move** | **0.750399** | **0.932096** |
| **# animals bought through market in the test-year from a farm, which was tested positive for TB the 24-12 months before the move** | **# animals bought through market in the test-year from a farm, which was tested negative for TB in the 36-24 months before the move** | **0.762626** | **0.93982** |
| **Log_e_(# Calves born, previous year)** | **Log_e_(mean herd size, in that year)** | **0.765087** | **0.705649** |
| **FMD Indicator 1; = 1 post February 2001** | **FMD Indicator 2; = 1 post February 2002** | **0.797681** | **0.797681** |
| **Depopulation indicator = 1 if herd depopulated in the past** | **Log_e_ # animals bought directly in the previous test-year from a farm, which always tested negative for TB before the move** | **0.803316** | **0.687473** |
| **Log_e_(# Calves born, previous year)** | **Log_e_(# Calves born, two years previously)** | **0.826907** | **0.816047** |
