# Supplementary material for: Impact of Imperfect Test Sensitivity on Determining Risk Factors: The Case of Bovine Tuberculosis
Source: PLoS One. 2012 Aug 13;7(8):e43116. doi: 10.1371/journal.pone.0043116 (PMC3418251; doi:10.1371/journal.pone.0043116)
Supplement: Appendix S1 — MCMC algorithm. Expression of the likelihood for discrete time survival model with multiple patterns. (DOCX) [file pone.0043116.s002.docx]

Appendix S1: MCMC algorithm

**Expression of the likelihood for discrete time survival model with multiple patterns.**

In the general case of assuming a 2-level random intercept model with potential multiple patterns for each herd, the MCMC algorithm has the following four steps with the choice of using a Metropolis or Gibbs steps for the pattern selection.

Let us assume we have K geographic areas (occupiers) onto which are mapped *J_k_* herds each with *n_j_* records, then we can write the full joint posterior distribution as

$p(\beta)p(\sigma_{u}^{2})\prod_{k} \prod_{j}^{J_{k}} p(\theta_{j})p(u_{k}|\sigma_{u}^{2})\prod_{p} \prod_{i}^{n_{j}^{(p)}} L(y_{ij}^{(p)}|X_{ij}^{(p)},\beta,u_{k})^{I(\theta_{j}=p)}$Here *θ_j_* is the index for the pattern chosen from those available for herd *j* noting that some herds will have a fully observed pattern and hence *θ_j_* is redundant and will simply be the observed pattern. As each pattern will be potentially of a different length due to varying times at risk we define *n_j_*^(^*^p^*^)^ to be the length of pattern *p* for herd *j*. In practice, to avoid patterns for the same herd *j* having different length, when the herd is not at risk, the observed response was set to a different value (4; with a corresponding time at risk variable set to 0) and ignored in the computation of the likelihood.

Our MCMC algorithm has four sets of parameters to update: the pattern indicators, *θ_j_*, the fixed effects, *β*, the non-spatial random effects *u_k_* and their variance *σ^2^_u_*.

Step 1 *θ_j_*

Here the conditional posterior simplifies to

$$p(\theta_{j})\prod_{p} \prod_{i}^{n_{j}^{(p)}} L(y_{ij}^{(p)}|X_{ij}^{(p)},\beta,u_{k})^{I(\theta_{j}=p)}$$

and we can use either a Gibbs sampling step as θ_j_ will have a categorical posterior distribution or a Metropolis sampling step choosing the pattern to evaluate from the other potential patterns for that herd by drawing it at random from the set of potential patterns. In the case of the imperfect sensitivity testing, we used a Metropolis sampling step, where the proposed pattern is chosen based on the prior probability distribution of the patterns so that the prior and the Hastings ratio cancel.

Step 2 *β*

Here we can condition on the currently chosen patterns and so we get

$$p(\beta)\prod_{j} \prod_{i}^{n_{j}^{(\theta_{j})}} L(y_{ij}^{(\theta_{j})}|X_{ij}^{(\theta_{j})},\beta,u_{k})$$

i.e. when we know the pattern we have then identified the correct data and this step is as for a model with no missing data – typically a random walk Metropolis step.

Step 3 *u_k_*

Here again we can condition on the currently chosen patterns and so we get

$$p(u_{k}|\sigma_{u}^{2})\prod_{i}^{n_{j}^{(\theta_{j})}} L(y_{ij}^{(\theta_{j})}|X_{ij}^{(\theta_{j})},\beta,u_{k})$$

i.e. when we know the pattern we have then identified the correct data and this step is as for a model with no missing data – typically a random walk Metropolis step.

Step 4 *σ^2^_u_*

Here we have the typical posterior distribution for the variance which, assuming conjugate priors, is updated via Gibbs sampling

$$p(\sigma_{u}^{2})\prod_{k} p(u_{k}|\sigma_{u}^{2})$$
